# Cryobiopsy in the diagnosis of bronchiolitis: a retrospective analysis of twenty-three consecutive patients

**DOI:** 10.1038/s41598-020-67938-y

**Published:** 2020-07-02

**Authors:** Syakirin Sirol Aflah Syazatul, Sara Piciucchi, Sara Tomassetti, Claudia Ravaglia, Alessandra Dubini, Venerino Poletti

**Affiliations:** 1 Department of Diseases of the Thorax, G.B. Morgagni Hospital, Forlì, Italy; 2 Institute of Respiratory Medicine, Kuala Lumpur, Malaysia; 3 Radiology Department, G.B. Morgagni Hospital, Forlì, Italy; 4 Department of Experimental and Clinical Medicine, Careggi University Hospital, Florence, Italy; 5 Pathology Department, G.B. Morgagni Hospital, Forlì, Italy; 6 Department of Respiratory Diseases and Allergology, Aarhus University Hospital, Aarhus, Denmark

**Keywords:** Diseases, Chronic obstructive pulmonary disease

## Abstract

Bronchiolitis manifests as a variety of histological features that explain the complex clinical profiles and imaging aspects. In the period between January 2011 and June 2015, patients with a cryobiopsy diagnosis of bronchiolitis were retrospectively retrieved from the database of our institution. Clinical profiles, imaging features and histologic diagnoses were analysed to identify the role of cryobiopsy in the diagnostic process. Twenty-three patients with a multidisciplinary diagnosis of small airway disease were retrieved (14 females, 9 males; age range 31–74 years old; mean age 54.2 years old). The final MDT diagnoses were post-infectious bronchiolitis (n = 5), constrictive bronchiolitis (n = 3), DIPNECH (n = 1), idiopathic follicular bronchiolitis (n = 3), Sjogren’s disease (n = 1), GLILD (n = 1), smoking-related interstitial lung disease (n = 6), sarcoid with granulomatous bronchiolar disorder (n = 1), and subacute hypersensitivity pneumonitis (n = 2). Complications reported after the cryobiopsy procedure consisted of two cases of pneumothorax soon after the biopsy (8.7%), which were successfully managed with the insertion of a chest tube. Transbronchial cryobiopsy represents a robust and mini-invasive method in the characterization of small airway diseases, allowing a low percentage of complications and good diagnostic confidence.

## Introduction

Bronchioles are non-cartilaginous small conducting airways with an internal diameter of approximately 2 mm^1^. Several classification schemes for bronchiolitis have been presented in the literature. In recent years, Ryu et al. ^2^ differentiated bronchiolar diseases into three groups: primary bronchiolar disorders, interstitial lung disease with prominent bronchiolar involvement and bronchiolar involvement in large airway disease.

The first category includes those entities in which bronchiolar disorder is the predominant abnormality, such as constrictive bronchiolitis, acute bronchiolitis, diffuse panbronchiolitis, respiratory bronchiolitis, mineral dust airway disease, and follicular bronchiolitis.

The second group represents all parenchymal lung diseases that can affect bronchioles to varying degrees, such as hypersensitivity pneumonitis, RB-ILD and DIP.

The third group represents the bronchiolar involvement of large airway disease, as in bronchiectasis, COPD or asthma.

There are several causes of bronchiolar diseases^2^: infections, inhalation of toxic agents (toxins, dusts or gases), drug reactions, collagen vascular disease, graft versus host disease (GVHD), lung transplantation, chronic occult aspiration, inflammatory bowel disease (IBD), and constrictive bronchiolitis associated with paraneoplastic autoimmune multi-organ syndrome^3–6^.

Even a preneoplastic disorder, diffuse idiopathic neuroendocrine cell hyperplasia with constrictive bronchiolitis (DIPNEH), has been included in this category^8,9^.

In a minority of cases, the aetiology of bronchiolar disorders remains unknown^10–22^.

A fundamental step for diagnosis and classification is the computed tomography (CT) scan^23–26^. The secondary lobule is the smallest unit that can be imaged; it has a polygonal shape, measuring approximately 2 cm in size. Inside the secondary lobules, there are 6 to 8 primary lobules with a central terminal bronchiole, with further subdivision into pyramidal acini, whose apices are located halfway between the centre of the secondary lobule and the interlobular septa. In this anatomical context, radiological signs of bronchiolar disorders can be directly identified (direct signs) or indirectly inferred (indirect signs). Therefore, ill-defined ground-glass centrilobular nodules are related to filling of the centrilobular space; tree-in bud patterns are related to mucoid impaction of the terminal bronchioles, and mosaic attenuation and air trapping in the expiratory scans are related to the obstruction of terminal bronchioles and secondary vasospasm^27–36^.

Even though surgical lung biopsy is still considered the gold standard for diagnosis, transbronchial cryobiopsy (TBLC) is an emerging tool that is being considered as a valid alternative, as it has already been demonstrated for fibrosing and non-fibrosing ILDs^37–50^. The aim of this article was to evaluate the role of cryobiopsy in the diagnostic work-up of bronchiolar disorders.

## Methods

### Patients

The study was approved by the USL Romagna-Ethical Committee as part of the study on cryobiopsy feasibility: prior to the procedure, written informed consent was obtained from all patients. Moreover, all methods were performed in accordance with the relevant guidelines and regulations of the journal.

We retrospectively retrieved from the database of our institution patients who underwent transbronchial cryobiopsy and who subsequently received a multidisciplinary diagnosis of bronchiolitis between January 2011 and June 2015.

The inclusion criteria were a histologic diagnosis through cryobiopsy and the availability of CT images.

### Radiological assessment

A CT scan (16-slice Light Speed scanner GE Medical Systems, Milwaukee, WI) was performed at least one month before biopsy in all cases. The parameters of acquisition were as follows: 1.5-mm slice thickness with 1-mm collimation and 1-mm reconstruction. The expiratory scan was performed in each patient for the entire lung at 10–20 mm intervals. All images were reviewed at window settings optimized for the lung parenchyma (width, 1,500–1,600 HU; level, 2,500 to 2,600 HU).

Findings were recorded according to the Fleischner Nomenclature^51^ as follows: ill-defined centrilobular nodules, tree-in-bud patterns, mosaic attenuation, and air trapping. Furthermore, ancillary and incidental findings were recorded.

### Biopsy procedure

A flexible cryoprobe measuring 90 cm in length and 1.9 mm in diameter was used (ERBE, Germany). The patients were deeply sedated with intravenous propofol and remifentanil and, while spontaneously breathing, intubated with a rigid tracheoscope. The biopsies were obtained under fluoroscopic guidance using the flexible bronchoscope inserted through the rigid tube into the selected bronchus. A distance of approximately 10 mm from the thoracic wall was considered optimal and always assessed by fluoroscopy. Once brought into position, the probe was cooled for approximately 7–8 s; then, it was retracted with the frozen lung tissue attached to the tip of the probe. The frozen specimen was thawed in saline and then transferred to formalin for fixation. Four specimens from at least two different segments from the same lobe were obtained.

A Fogarty balloon was prophylactically placed in the segmental bronchus near the biopsy site and routinely inflated after sampling to minimize the consequences of haemorrhage. The choice of the site and side of biopsy was decided upon before the procedure on the basis of the distribution of CT findings.

### Histologic analysis

Haematoxylin–eosin–stained slides were reviewed, and specific stains were obtained when deemed useful. Histologic diagnosis of bronchiolitis was based on the above-mentioned classifications^2,3^. Specimens were reviewed by two lung pathologists (AD and VP).

## Results

### Patients

Twenty-three patients with an MDT diagnosis of bronchiolar disease (9 males, 14 females; age range 37–74 years old; mean age 54.2) were collected.

After the procedure, two subjects (8.7%) developed pneumothorax, which was successfully managed with chest tube insertion. No complications were reported.

The histologic categories identified were grouped according to the Ryu classification into primary bronchiolitis and interstitial lung diseases with a prominent bronchiolar component. No cases of bronchiolar disorders associated with large airway disease underwent a cryobiopsy procedure.

In the primary bronchiolitis section, ten patients showed cellular bronchiolitis (5 cases of post-infectious bronchiolitis and 5 cases of follicular bronchiolitis), and three cases showed constrictive bronchiolitis (2 cases of cryptogenic bronchiolitis and 1 case of DIPNECH).

In the group of ILDs with prominent bronchiolar components, we recorded 6 cases of RB-ILD, 1 case of granulomatous bronchiolitis and 2 cases of hypersensitivity pneumonitis.

Age, sex, clinical symptoms, and smoking status are summarized in Tables 1–2. The imaging findings are summarized in Table 3.

**Table 1:** Demographic and clinical features of patients with primary bronchiolar disorder.

	Cellular Bronchiolitis		Constrictive Bronchiolitis	
	Infectious Bronchiolitis (n = 5)	Follicular Bronchiolitis (n = 5)	Cryptogenic Constrictive Bronchiolitis (n = 3)	DIPNECH (n = 1)
Age – yrs (range)	41–70	31–68	48–61	74
Sex	4 F, 1 M	4 F, 1 M	1 F, 2 M	1 F
Smoking status	Current smoker (n = 1) Former smoker (n = 2)	Former smoker (n = 3)	Current smoker (n = 1)	Former smoker
Symptoms	Cough (n = 5) Dyspnoea (n = 3) Fever (n = 4)	Cough (n = 3) Dyspnoea (n = 1) Fever (n = 2)	Cough (n = 1) Dyspnoea (n = 1) Fever (n = 1)	Cough

**Table 2:** Demographic and clinical features of patients with ILD with a prominent bronchiolar component.

	Respiratory Bronchiolitis-ILD (n = 6)	Granulomatous Bronchiolitis (n = 1)	Hypersensitivity Pneumonitis (n = 2)
Age – yrs (range)	37–62	52	63
Sex	2 F, 4 M	M	1 M
Smoking status	Current smoker (n = 6)	Non-smoker	Former smoker
Symptoms	Cough (n = 3) Dyspnoea (n = 3) Fever (n = 2)	Dyspnoea	Cough Fever Dyspnoea

**Table 3:** Imaging findings for each histotype. Infectious bronchiolitis was mainly represented by a tree-in-bud pattern. Follicular bronchiolitis was characterized by ill-defined centrilobular nodules and ground-glass attenuation. Constrictive bronchiolitis was characterized by tree-in bud patterns and mosaic attenuation. DIPNECH was characterized by the coexistence of nodules and mosaic attenuation. In HP, ill-defined centrilobular nodules were associated with air trapping and ground-glass attenuation. Granulomatous bronchiolitis was characterized by ill-defined nodules.

	Cellular Bronchiolitis		Constrictive Bronchiolitis		ILD with prominent bronchiolar disorder		
CT findings	Infectious Bronchiolitis N (%)	Follicular Bronchiolitis N (%)	Cryptogenic Constrictive Bronchiolitis N (%)	DIP-NECH N (%)	HP N (%)	RB-ILD N (%)	Granul N (%)
Tree-in-bud	5 (100%)	2 (40%)	2 (100%)			1 (16%)	
Free-standing Bronchiectasis	2 (40%)	1 (20%)					
Ill-defined nodules	2 (40%)	1 (20%)			1 (50%)	5 (83%)	1 (100%)
Ground-glass attenuation	1 (20%)	2 (40%)			1 (50%)	1 (16%)	
Air trapping		1 (20%)	1 (50%)	1 (100%)	1 (50%)	1 (16%)	
Solid nodules		1 (20%)		1 (100%)			
Nodules with halo sign		1 (20%)					

### Primary Bronchiolar disorders

**Figure 1:**
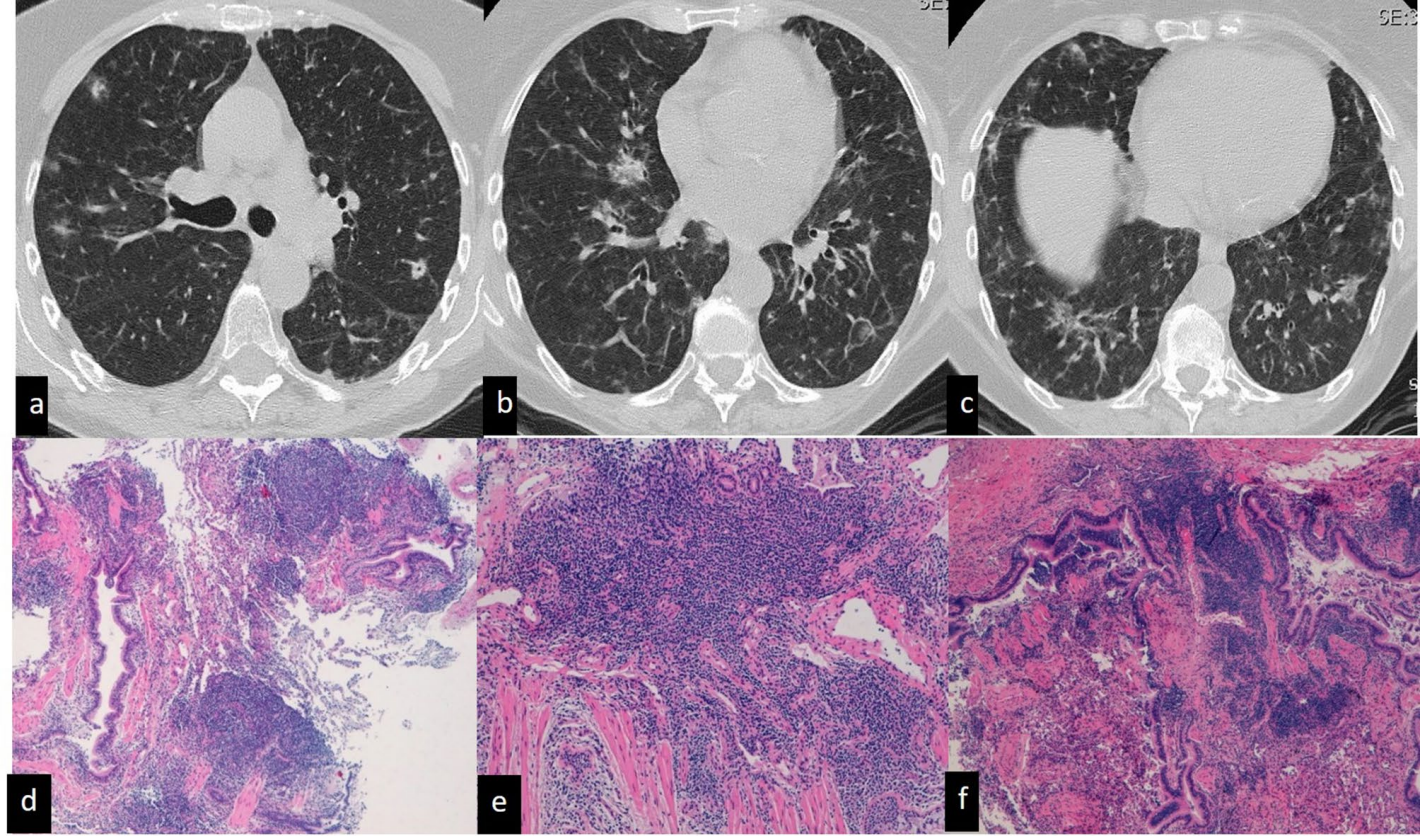
Idiopathic follicular bronchiolitis. CT scan (**a**–**c**) shows multiple bilateral nodules and round consolidations, some of which have halo signs, mainly along the bronchovascular bundle in the middle lobe, right and left lower lobes and apico-dorsal segment of the left upper lobe. Histopathological examination shows that the bronchiole is surrounded and infiltrated by lymphoid aggregates.
  Treatment consisted of steroids in cases of an idiopathic form and rituximab, azathioprine and immunoglobulins in the case of GLILD.

**Figure 2:**
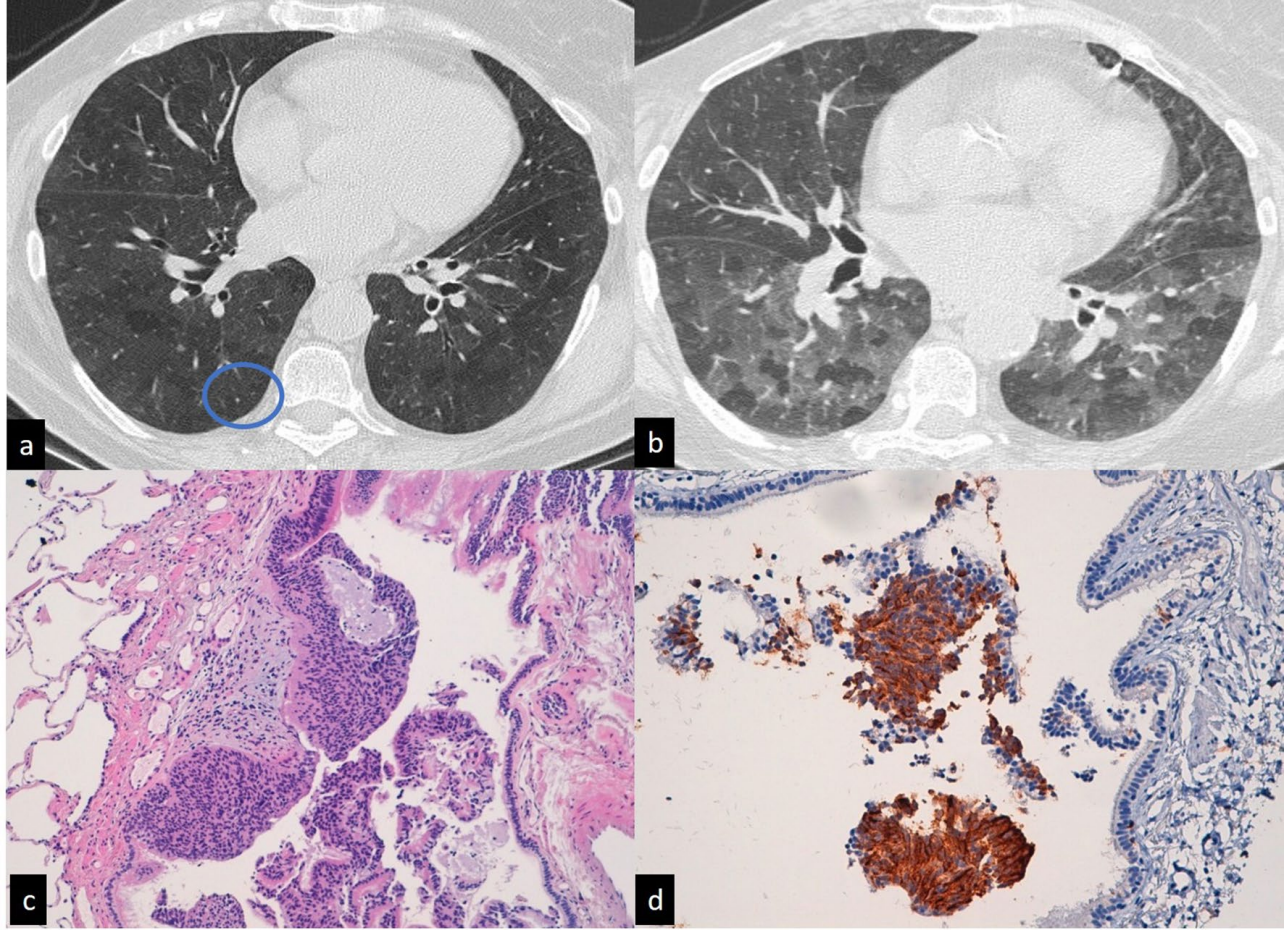
Diffuse idiopathic pulmonary neuroendocrine hyperplasia (DIPENCH). CT scan (**a**, **b**) shows diffuse mosaic attenuation in both hemithoraces. A tiny nodule (**a**, circle) is also present in the right lower lobe. In the expiratory scan (**b**), diffuse air trapping can be observed. Histopathological specimens (**c**, **d**) show bronchioles obliterated by the nodular proliferation of neuroendocrine cells.

- **Cellular Bronchiolitis**
- Infectious Bronchiolitis: five patients were recorded (4 females, 1 male). Cultures of the bronchoalveolar lavage fluid were positive for *Haemophilus influenza* in 3 patients, *Nocardia abscess* in one case and Mycobacterium avium-intracellular complex in one case. Samples were characterized by the presence of debris, neutrophil micro-abscesses and submucosal oedema.
  In all cases, CT scans showed a prominent tree-in-bud pattern related to mucoid impaction of the terminal bronchioles. Mild concomitant mosaic attenuation was observed.
  All the patients received antibiotic therapy after MDT diagnosis.
- Follicular Bronchiolitis: five patients were recorded (4 females, 1 male). Histologically, follicular bronchiolitis was characterized by the presence of lymphoid follicles with germinal centres around the small airways. In all cases, the microbiological investigation results were negative.
  CT scan features included the following: ill-defined nodules in one case, ground-glass attenuation and nodules with halo signs in one case, tree-in-bud patterns in two cases, and mosaic attenuation in one case.
  The final MDT diagnoses were as follows: Sjogren’s syndrome in one case, an idiopathic form in three cases (Fig. 1), and GLILD in one case affected by common variable immunodeficiency.

- **Constrictive Bronchiolitis**
- Three patients: the CT scan findings were tree-in-bud patterns in two cases and mosaic attenuation and air trapping in one case. Treatment consisted of antibiotics and immunomodulators for the cryptogenic forms.
- Diffuse Idiopathic Pulmonary Neuroendocrine Cell Hyperplasia (DIP-NECH): one case. The patient presented with typical CT findings: mosaic attenuation and air trapping as well as small scattered nodules (Fig. 2). Treatment consisted of follow-up.

### ILD with a prominent bronchiolar component

- **Respiratory Bronchiolitis-ILD** (RB-ILD) six patients (2 females, 4 males); all the subjects were current smokers. CT scans showed ill-defined ground-glass nodules in five cases representing RB-ILD and diffuse ground-glass attenuation in one case mostly related to desquamative interstitial pneumonia (DIP). This patient had significant eosinophilia in the bronchoalveolar lavage (BAL) fluid, and his final diagnosis was DIP.
- **Hypersensitivity Pneumonitis (HP)** In two patients, the final diagnosis was chronic hypersensitivity pneumonitis. In one case, a positive history of exposure to birds and positive serum precipitins were confirmed. In the second subject, the final diagnosis was subacute hypersensitivity pneumonitis related to sulphasalazine treatment. CT scan aspects were mosaic attenuation and centrilobular nodules. Treatment consisted of drug suspension.
- **Granulomatous** In one case, a peculiar clinical background of sarcoidosis and Evan's syndrome was identified. CT scans were characterized by ill-defined nodules. Histology showed small non-necrotizing granulomas around the small airways. The final diagnosis was granulomatous bronchiolitis in concomitant sarcoidosis, and treatment consisted of steroids and rituximab.

## Discussion

Small airway disease, or bronchiolitis, is a broad term encompassing numerous diseases that cause bronchiolar inflammation or fibrosis. It generally refers to airways measuring less than 2 mm in diameter, lacking the cartilaginous component in the wall of the airway until the alveolar ducts^52–53^. This heterogeneous group usually manifests with non-specific respiratory symptoms such as cough, dyspnoea and fever. Imaging can show a variety of findings, depending on the physiopathology present in the background.

As the smallest unit imaged on CT scans is the secondary lobule, the direct signs of bronchiolitis include centrilobular nodules or centrilobular ground-glass opacities and a tree-in-bud pattern typically reflecting a cellular or inflammatory form of bronchiolitis.

Indirect signs are air trapping and mosaic attenuation. Air trapping results from the partial outflow obstruction of the small airways. This can be related to a collapse of the bronchioles during expiration. Air trapping is characterized by areas of low attenuation of the pulmonary density and is a sign of constrictive bronchiolitis^52^.

Mosaic attenuation can be attributed to vascular causes as well as to bronchiolitis. In small airway diseases, such as bronchiolitis, a vasoconstrictive reflex can be observed, resulting in hypoperfusion^53^.

Diagnosis requires the combination of clinical, radiological and histological data^54–60^.

In our cohort of patients, a tree-in-bud pattern, as a direct sign of bronchiolar mucous impaction, was mostly represented in the group of infectious bronchiolitis (5 cases), follicular bronchiolitis (2 out of 5) and constrictive bronchiolitis (2 out of 3).

Ill-defined centrilobular nodules were the most frequent in the group of RB-ILD cases (5 out of 6) and were seen in a case of HP.

Mosaic attenuation and air trapping were mostly represented in the group of constrictive bronchiolitis (1 case), DIPNECH (1 case) and bronchiolitis in HP (1 case).

Traditional transbronchial biopsy has been recently investigated for its accuracy in the diagnosis of interstitial lung disease and has demonstrated an accurate diagnosis in only 20% to 30% of cases. Therefore, a surgical lung biopsy is still considered crucial to provide large and diagnostic specimens^60^.

In recent years, transbronchial cryobiopsy has shown a high diagnostic yield in diffuse parenchymal lung disorders in contrast to conventional forceps biopsy with a diagnostic accuracy closer to that of surgical lung biopsy^61^. Recently, Colella et al.^62^ emphasized the diagnostic value of TBLC in evaluating subsets of asthmatic patients. They, in fact, performed cryobiopsy in three patients affected by asthma, obtaining useful information for the pathophysiology of the disease. With TBLC samples, the authors obtained information about bronchiolar changes, particularly goblet metaplasia and eosinophilic infiltration of mucosa and submucosa of bronchioles and nodular lymphoid inflammation.

Our investigation sought to evaluate whether TBLC combined with CT interpretation and multidisciplinary discussion could be a promising *and safe tool* comparable and an alternative to diagnosis with surgical biopsy, as recently stated by Hetzel and co-workers^63^.

**Figure 3:**
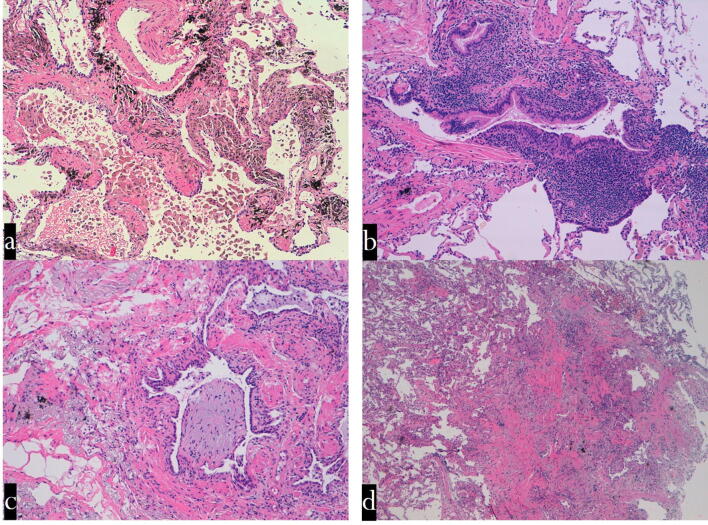
Transbronchial cryobiopsy. Histological findings (haematoxylin–eosin staining: mid power). (**a**) Respiratory bronchiolitis: a smoker's macrophages in the lumen of the respiratory bronchiole and surrounding alveoli. The wall of the respiratory bronchiole is thickened by collagen deposition. (**b**) Follicular bronchiolitis: lymphoid follicles are evident in the wall of a terminal bronchiole. The lumen of this airway is partly occluded. (**c**) ILD with prominent bronchiolar involvement: The lumen of a terminal bronchiole is almost completely occluded by a polyp made up mainly of loose connective tissue (bronchiolitis in HP). (**d**) Constrictive bronchiolitis: a terminal bronchiole is completely substituted by a scar. *HPE histopathological examination, GL ILD granulomatous lymphocytic interstitial lung disease, CVID common variable immunodeficiency, GGO ground-glass opacity, NSIP nonspecific interstitial pneumonia, ILD interstitial lung disease, ANCA—anti-neutrophilic cytoplasmic antibody, OP organizing pneumonia, COPD chronic obstructive pulmonary disease, MAC—mycobacterium avium complex, HP hypersensitivity pneumonitis, DIPNECH diffuse idiopathic pulmonary neuroendocrine cell hyperplasia, CT computed tomography, DIP desquamative interstitial pneumonia, ILD smoking-related interstitial lung disease.

Based on these data, the histological information from cryobiopsy specimens allowed an appropriate diagnosis of cellular, respiratory, and follicular bronchiolitis, as well as constrictive bronchiolitis (Fig. 3). Interestingly, cryobiopsy has been shown to be a valid tool in the diagnosis of pre-neoplastic conditions, such as DIPNECH sampling of small tumourlets and bronchiolar fibrosis.

In detailed cryobiopsy, samples are large enough for the identification of terminal and respiratory bronchioles and for retrieving a conspicuous amount of alveolar tissue. The literature indicates that the pneumothorax rate varies from less than 1% to almost 30%^55^.

Pneumothorax has been recorded as the main complication observed, but it was observed in a minority of cases (8.6%). Based on the reported data^63^, TBCB should be performed by interventional pulmonologists appropriately trained in a centre familiar with advanced therapeutic bronchoscopic procedures (management of massive haemoptysis and tension pneumothorax). There are two major limitations in this study that could be addressed in future research.

First, the analysis focused on a relatively small sample of patients; second, it was based on a retrospective observational analysis, which likely resulted in the loss of some indeterminate cases. Further prospective studies with a larger cohort of patients are recommended.

## Conclusion

TBCB is a promising technique that substantially expands the pulmonary armamentarium in the diagnosis of bronchiolitis.
